# Therapeutic Prospects of Abemaciclib for Patients with Endometrial Cancer

**DOI:** 10.3390/curroncol31090397

**Published:** 2024-09-12

**Authors:** Ahmad Awada, Sarfraz Ahmad

**Affiliations:** Gynecologic Oncology Program, AdventHealth Cancer Institute, Orlando, FL 32804, USA

**Keywords:** endometrial cancer, abemaciclib, cyclin-dependent kinase inhibitor, antitumor, clinical response, breast cancer

## Abstract

Endometrial cancer (EC) is a common gynecologic malignancy with a rising incidence due to obesity, comorbid conditions, and related lifestyle factors. The standard of care for primary disease consists of surgical resection with/without chemotherapy ± radiotherapy for select patients. Recurrence is common in patients with advanced-stage disease and/or high-risk features, who primarily are treated with systemic therapy. The identification of novel targets in malignant EC has led to the development of wide-range inhibitors. Abemaciclib is an orally active unique cyclin-dependent kinase (CDK) inhibitor, selective for the CDK4 and CDK6 cell cycle pathways. This agent has potential anti-neoplastic activity and is indicated in combination with various therapies such as endocrine therapy, aromatase inhibitors, and hormone therapies, primarily in breast cancer (BC). Herein, we sought to summarize the biochemical/pharmacological properties of abemaciclib and its therapeutic potential in EC. While the therapeutic role(s) of abemaciclib was fairly established in a subset of patients with advanced/metastatic BC through the pivotal MONARCH trials, its attributes and clinical utility in EC are limited. Thus, based on some promising pre-clinical/translational insights and a recent phase II study, we highlight abemaciclib’s properties and potential clinical usefulness in patients with EC, particularly in recurrent estrogen-receptor-positive cases.

## 1. Introduction/Background

Endometrial cancer (EC) is the most common gynecologic cancer, with estimates of about 67,880 new cases of uterine cancer and about 13,250 deaths in the year 2024 in the United States [1]. Accounting for histological and clinical behavior differences, EC has been categorized as type I or type II disease [2]. Type I disease, accounting for 80% of all EC cases, is hormonally driven with a favorable prognosis; it is typically estrogen-induced, responsive to progestin, and includes endometrioid subtypes. While type II disease consists of high-grade histologies (e.g., serous, clear cell, etc.), it is estrogen-independent, arising in the background of endometrial atrophy, and carries more adverse prognosis [3]. Recently, The Cancer Genome Atlas (TCGA) performed a genome-wide analysis of EC, which led to classification into four molecular subtypes with different prognostic outcomes: polymerase epsilon (POLE) “ultra-mutated”, microsatellite instability (MSI) “hypermutated”, copy number low or no specific molecular profile (NSMP), and copy number high or p53 mutated [4,5].

NSMP cancers typically express estrogen and/or progesterone receptors. While there is no widely accepted threshold for defining estrogen/progesterone receptor positivity in EC, studies indicate varying outcomes based on receptor expression levels. Cases with 0–10% expression had the poorest outcomes, with 5-year disease-specific survival (DSS) rates of 75.9–83.3%. Those with 20–80% expression showed intermediate outcomes, with survival rates of 93–93.9%. In contrast, those with cancers with 90–100% receptor expression had the most favorable prognosis, with 5-year survival rates of 97.8–100% [4,5].

EC is surgically staged as per the joint committee of the 2017 International Federation of Gynecology and Obstetrics (FIGO). Subsequently, the decision to proceed with post-operative adjuvant therapy is reliant on the disease recurrence risk, which, in turn, is characterized by age, tumor histology, stage of the disease, and other pathologic factors. Adjuvant treatment may be offered, such as radiation, chemotherapy, or a combination of both [6,7,8].

Following appropriate surgical staging, adjuvant treatment for EC, if needed, is based on the risk of disease recurrence, which is influenced by factors such as age, tumor histology, and disease stage and includes radiation, chemotherapy, or a combination of both. Additionally, in advanced-stage EC, the combination of platinum-based chemotherapy and immunotherapy was shown to improve progression-free survival (PFS) and overall survival (OS), particularly in an MSI-high subgroup [9].

For women with recurrent disease other than loco-regional recurrence, treatment is tailored based on whether the patient had prior chemotherapy. For chemotherapy-naïve patients, carboplatin and paclitaxel are the standard of care, with an objective response rate (ORR) of 50% and a progression-free survival (PFS) of 14 months [10]. For patients who had prior chemotherapy, treatment with an immune checkpoint inhibitor (ICI) is recommended if the tumor is mismatch-repair-deficient (dMMR) or the tumor mutation burden (TMB) is high. Otherwise, for mismatch-repair-proficient (pMMR) tumors, we offer a combination of pembrolizumab (an ICI) and lenvatinib (a tyrosine kinase inhibitor, TKI) based on the KEYNOTE-775 trial [11,12].

The treatment options for recurrent EC depend on the location of recurrence, prior treatments, and the patient’s overall health. Surgery may be an option for localized recurrences or metastases, particularly if radiation therapy has not been previously utilized. Radiation, including external beam radiation therapy (EBRT) or vaginal brachytherapy, can be effective for pelvic or vaginal recurrences. Chemotherapy, typically platinum-based agents such as carboplatin and paclitaxel, combined with ICIs (i.e., dostarlimab or pembrolizumab), is the first-line treatment, with significant benefit observed in MSI-high tumors. Additionally, chemotherapy can be paired with targeted therapies like bevacizumab, a vascular endothelial growth factor (VEGF) inhibitor. For MSI-high tumors, single-agent immunotherapy is viable, while the combination of pembrolizumab and lenvatinib is approved for tumors without these markers [13].

Alternatively, hormonal therapies, such as progestins, aromatase inhibitors, and tamoxifen, remain options for hormone-receptor-positive cancers with modest efficacy. Thus, many EC trials have explored targeted therapy in estrogen receptor (ER)-positive tumors based on established efficacy in hormone-receptor-positive breast cancer. Indeed, abemaciclib, a selective cyclin-dependent kinase 4/6 (CDK4/6) inhibitor, is being explored as a promising therapeutic option for EC, particularly for patients with dysregulated CDK4/6 signaling pathways. By inhibiting these kinases, abemaciclib prevents the phosphorylation of the retinoblastoma (RB) protein. Normally, CDK4/6 and cyclin D work together to phosphorylate RB, leading to the release of E2F transcription factors that drive cell cycle progression from the G1 phase to the S phase. By inhibiting CDK4/6, abemaciclib keeps RB bound to E2F, blocking E2F activity and thereby halting cell cycle progression. This results in cell cycle arrest and inhibits the proliferation of tumor cells, particularly those with dysregulated cyclin D-CDK4/6 activity [14].

Subsequent-line therapies are mainly palliative and include single-agent chemotherapy, combination chemotherapy, endocrine therapy, bevacizumab, or consideration of pathway-specific therapies in clinical trials.

## 2. Estrogen and EC

**Figure 1:**
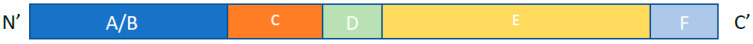
Estrogen receptor and its multiple domains. *Abbreviations:* A/B: Transcriptional activation function domain (AF-1); C: DNA-binding domain; D: hinge; E: ligand binding domain; F: transcriptional activation function domain (AF-2).

Estrogen in the cytoplasm passes through the cell membrane, which binds to the estrogen receptor (ER). Then, the ER dimerizes and translocates to the nucleus, where it promotes downstream DNA transcription into mRNA and, finally, into protein, resulting in a change in cell function. ER includes two different forms, i.e., ERα and ERβ, encoded by chromosomes 6 and 14, respectively [15]. Both receptors are composed of five domains [Figure 1]. ERα consists of two transcriptional activation function (AF) domains, viz., AF-1 and AF-2. While the AF-1 domain is localized in the N-terminal region, the balance of the transcriptional activation domains differs among tissues, with the dominance of the AF-2 domain in mammary gland cells and the AF-1 domain in endometrial cells [16].

**Figure 2:**
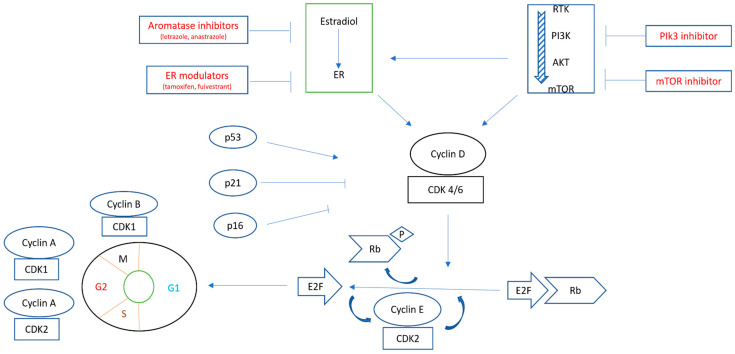
Schematic presentation of the interaction between estrogen receptor (ER) and the PI3K/AKT/mTOR pathway. They converge on activating CDK4/6, leading to phosphorylation of Rb and releasing E2F, which, in turn, allows the progression of the cell cycle from the G1 to the S phase. Multiple therapeutic agents can be used to block the steps of such pathways [17,18].

In EC, there is cross-regulation between ER and the intracellular pathway CDK-RB-E2F [cyclin-dependent kinase–retinoblastoma–E2F family of transcription factors], leading to cell cycle regulation. Also, E2 and insulin act synergistically, and their pathways converge on cyclinD-CDK4/6, leading to increased expression and stabilization of cyclin D, thus activating CDK4/6, which in turn phosphorylates RB (retinoblastoma) and releases E2F, leading to cell cycle progression to the S phase. Also, E2 is a potential activator of the mitogen-activated protein kinase (MAPK) pathway through ER and insulin receptor (InsR) interaction. Synergistically, insulin with ER activates the phosphorylation of MAPK and phosphatidylinositol 3-kinase (PI3K pathways) [Figure 2] [17,18].

## 3. CDK-RB Pathway in EC

**Figure 3:**
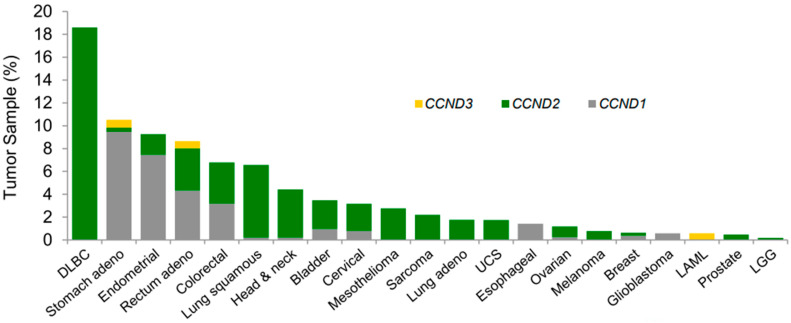
Genomic aberrations that activate D-type cyclins are associated with enhanced sensitivity to the CDK4 and CDK6 inhibitor (abemaciclib) [19]. Frequency of 30 UTR loss in the mRNA for *CCND1*, -*2*, or -*3* across TCGA tumor samples. *Abbreviations:* CDK, cyclin-dependent kinase; TCGA, The Cancer Genome Atlas; LAML, acute myeloid leukemia; LGG, lower-grade glioma; UCS, uterine carcinosarcoma.

D-cyclin activating features (DCAFs) are defects that lead to the inappropriate expression of D-type cyclins. DCAFs are associated with *CCND1* gene translocation, *CCND1-3* 3′UTR loss, *CCND2* or *CCND3* amplification, and K-cyclin and *FBXO31* loss. Actually, based on TCGA data, 5% of previously untreated EC bears a *CCND1* 3′UTR mutation; in addition, 16% of EC has cyclin D1 aberration [Figure 3]. Moreover, DCAFs are associated with high sensitivity to CDK4/6 inhibitors [19].

**Figure 4:**
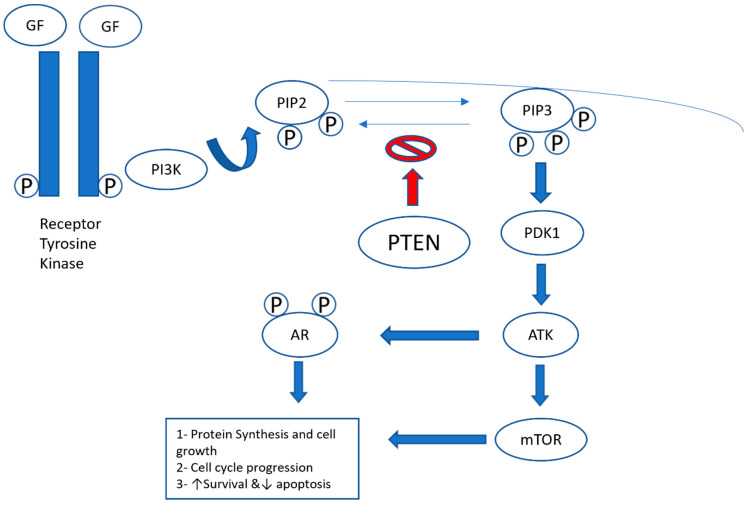
PI3K (phosphatidylinositide 3-kinases)/PTEN (phosphatase and tensin homolog gene)/Akt pathway. The binding of growth factors (GFs) to the receptor tyrosine kinase activates the receptor complex, which, in turn, recruits and activates PI3K. The activated PI3K converts PIP2 (phosphatidylinositol-4,5-diphosphate) to PIP3 (phosphatidylinositol-3,4,5-triphosphate), which subsequently mediates the phosphorylation of Akt through PDK1 (protein kinase 1). Phosphorylated Akt is active on a wide range of substrates, but one of its most important targets is mTOR, which is involved in cell growth, proliferation, and survival. Activated Akt (protein kinase B) also interacts with the androgen receptor (AR) in an androgen-independent manner, leading to overactivation of the AR signaling pathway in castration-resistant prostate cancer. PTEN is a tumor suppressor that negatively regulates the pathway by removing the 3-phosphate from PIP3, converting it back to PIP2. Loss of PTEN leads to overactivation of Akt, which, in turn, is associated with uncontrolled cell proliferation, decreased apoptosis, and enhanced tumor angiogenesis [22].

Mutation of the tumor suppressor gene [e.g., phosphatase and tensin homolog (PTEN)] is seen in 80% of EC cases [20]. PTEN loss results in cell division dysregulation and the accumulation of cyclinD1-CDK4/6, which potentially makes it a target for CDK4/6 inhibitors. Dosil et al. [21] showed an important role of cyclinD-CDK4/6 in the development of PTEN-driven EC [22], as we schematically illustrate in Figure 4.

Semczuk et al. [23] showed that 69% of ECs demonstrate aberrant expression of at least one of the retinoblastoma-pathway proteins, in addition to CDK4 overexpression being associated with disease progression. Hu et al. [24] reported that CDK4 was overexpressed in EC tissues and demonstrated a positive correlation with activator E2F transcriptional factors activity. This observation indicates that CDK4 inhibition may offer a rationale and an effective treatment strategy for patients with advanced-stage EC.

## 4. Endocrine Therapy in EC

**Figure 5:**
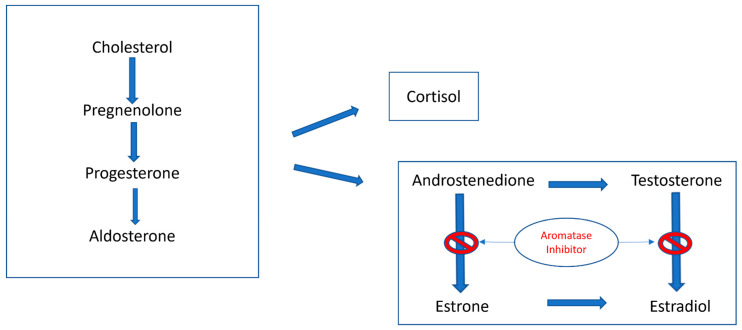
Schematic presentation of steroidogenesis showing the role of aromatase inhibitors in blocking the conversion of androstenedione and testosterone to estrone and estradiol, respectively. There are different types of aromatase inhibitors: Type I steroidal drugs bind competitively but irreversibly to the enzyme, while Type II non-steroidal inhibitors, such as anastrozole and letrozole, bind reversibly to the enzyme and fit into the substrate binding site [27].

Endocrine therapy remains an attractive therapeutic strategy due to its favorable toxicity profile and administration mode. However, the currently available hormonal therapies yield only modest results. In the GOG-81 study, 145 patients with recurrent EC received 200 mg of medroxyprogesterone PO daily with an ORR of 25% [17% complete response (CR), 8% partial response (PR)], and the median-PFS (mPFS) was 3.2 months [25]. In the GOG-153 phase II trial of alternating courses of megestrol acetate (80 mg BID × 3 weeks) and tamoxifen (20 mg BID × 3 weeks) in 56 patients with advanced EC, the ORR was 27% (21% CR, 6% PR), and the mPFS was 2.7 months, but in 53% of the responders, the duration of response surpassed 20 months. Letrozole and anastrozole, aromatase inhibitors [Figure 5], when evaluated in a phase II trial of women with persistent or advanced-state EC, showed an ORR < 10%, yet when combined with everolimus, a mammalian target of rapamycin (mTOR) inhibitor, led to an ORR of around 32% [26].

## 5. CD4/6 Inhibitor

It has been shown that endometrioid ECs exhibit phosphoinositide 3-kinase (PI3K) pathway and receptor tyrosine kinase (RTK)/RAS/b-catenin (CTNNB1) pathway alterations in 90% and 80% of cases, respectively [20]. Both pathways are linked to endocrine therapy resistance as they facilitate ligand-independent activation of ER transcriptional activity and promote ER-independent upregulation of cyclin D1 (*CCND1*), a known ER target gene crucial for estrogen-induced cell proliferation. In addition, the PI3K pathway and RTK/RAS/CTNNB1 pathways upregulate *CCND1*, which activates CDK4 and CDK6, leading to the phosphorylation of retinoblastoma (RB1) and its separation from transcription factor E2F, facilitating the G1 to S phase transition [28,29].

CDK4/6 inhibitors have proven potent antitumor activity, for example, in estrogen/progesterone+/HER2− breast cancer patients. The CDK4/6 genes (and some other related genes) are widely expressed in several tumors (as shown by TCGA database analyses), and when moderately–highly expressed, they indicate poor survival; thus, the most effective treatment strategy might be a combination therapy consisting of CDK4/6 inhibitors with other signaling molecule inhibitors [30].

## 6. CD4/6 Inhibitors in Breast Cancer

Among patients with breast cancer, approximately 75–80% of cases are hormone receptor (HR)-positive, for which endocrine therapy is the main therapy, yet drug resistance tends to become unavoidable in the course of treatment. Combining endocrine therapy with chemotherapy yielded modest efficacy due to the relatively low survival benefits and relatively higher toxicity in patients with HR-positive breast cancer. Thus, clinicians are encouraged to investigate improved endocrine therapy efficacy approaches [31].

The MONARCH-1 trial studied abemaciclib, a CDK4/6 inhibitor, in 132 patients with refractory HR+/HER2− breast cancer and demonstrated an ORR of 19.7%, mPFS of 6.0 months, and mOS of 17.7 months [32]. This was followed by the MONARCH-2 trial, which showed that abemaciclib with fulvestrant [ER antagonist and estrogen receptor degrader (SERD)] vs. fulvestrant in the same cohort population as MONARCH-1 had an mPFS of 16.4 months vs. 9.3 months (HR 0.55, *p* < 0.001) [33]. The MONARCH-3 trial compared abemaciclib + non-steroidal aromatase inhibitor vs. non-steroidal aromatase inhibitor (as first-line therapy in HR+/HER2− post-menopausal patients), reporting an mPFS NR vs. 14.7 months and an ORR 59% vs. 44%, both statistically significant [34]. Table 1 summarizes the reported key adverse events in treatment with abemaciclib for patients with breast cancer in the MONARCH studies noted above.

## 7. Biochemical and Pharmacologic Properties of Abemaciclib

Abemaciclib (VERZENIO^®^; Eli Lilly & Co., Cambridge, MA, USA) is an oral drug (a unique cyclin-dependent kinase inhibitor) with a molecular formula of C27H32F2N8 and a molecular weight of 506.6 Dalton (PubChem CID: 46220502). The compound is canonized, and its International Union of Pure and Applied Chemistry (IUPAC) name is N-[5-[(4-ethylpiperazin-1-yl)methyl]pyridin-2-yl]-5-fluoro-4-(7-fluoro-2-methyl-3-propan-2-ylbenzimidazol-5-yl)pyrimidin-2-amine. Abemaciclib is specifically based upon a distinct 2-anilino-2,4-pyrimidine-[5-benzimidazole] scaffold [35]. It has potential anti-neoplastic activity and is indicated for use in combination with various therapies such as endocrine therapy, aromatase inhibitors, and hormone therapies (primarily in breast cancer). The U.S. FDA approved abemaciclib for the treatment of patients with hormone-positive and HER2-negative advanced/metastatic breast cancer who progressed after endocrine therapy failed. Being effective at relatively low dosages with suitability for long-term administration, this agent has also been studied in various trials related to melanoma, lymphoma, neoplasm, solid tumors, and glioblastoma with limited scope [36].

## 8. Abemaciclib in EC

The molecular and genomic characteristics of EC help predict prognosis and response to therapy. As compared to normal endometrium, the mRNA expression along with transcriptional activity of E2Fs are increased in the tissues of patients with endometrial cancer. Moreover, CDK4 is highly expressed in EC tissues and correlates with increased E2F activity, suggesting that CDK4 inhibition may also offer an effective treatment option for advanced-stage EC patients. The study demonstrated that EC could be one of the sensitive tumor types, as CDK4 was shown to have higher expression compared to CDK6 in various reproductive organ systems, where abemaciclib has a higher potency against CDK4 compared to CDK6 [37].

CDK4/6 inhibitors (along with inducing cell cycle arrest) were proven to increase the breast cancer cell presentation of neoantigen and major histocompatibility complex (MHC) molecules by promoting interferon (IFN) activity, hence could promote antitumor immunity and enhance the action of ICI [24]. ICIs have already been established as effective adjuvant therapy for recurrent EC as a single agent or when combined with lenvatinib in dMMR or pMMR cases, respectively [11].

Indeed, pre-clinical trials related to abemaciclib with or without endocrine therapy have been tested on EC cell lines: HEC-1A cells (ER-negative, moderately differentiated endometrioid endometrial adenocarcinoma) and Ishikawa cells (ER-positive, well-differentiated endometroid endometrial adenocarcinoma) [36]. The combination of CDK4/6 inhibitors and AI is proven to induce a potent synergistic effect independent of ER expression [38]. Most recently, Konstantinopoulos et al. [39] reported phase II data from a two-stage study where letrozole/abemaciclib was given to 30 patients with recurrent ER-positive EC. The ORR was 30% (and 30% had CR), all in endometrioid adenocarcinomas. The reported median PFS was 9.1 months, whereas the PFS at 6 months was 55.6%, with the median duration of response (DOR) being 7.4 months [39].

## 9. Safety of Abemaciclib

In Konstantinopoulos et al.’s [39] phase II trial, patients received abemaciclib 150 mg p.o. twice daily, and letrozole at 2.5 mg daily. Treatment-related adverse events (TRAEs) were noted in 20% of patients. The most common grade (Gd) 3 TRAEs were neutropenia (n = 6; 20%) and anemia (n = 5; 17%). No treatment-related death was reported. Diarrhea was noted in 21 (70%) cases. It is known that abemaciclib inhibits renal transporters (such as OCT2, MATE1, and MATE2-K), and 33% of patients had reversible Gd1 or Gd2 increases in creatinine that led to no dose reduction or interruption. Patients (n = 16; 53%) showed one dose reduction of abemaciclib, which was the most common for diarrhea (n = 7) and fatigue (n = 6). Two (7%) cases discontinued therapy due to toxicity [39].

In the MONARCH trials, the following adverse events (AEs) were reported: *diarrhea*, which occurred within the first 7 days of abemaciclib treatment, which resolved within 2 weeks of anti-diarrheal medication/dose adjustments; *neutropenia*, occurring within the first two cycles of abemaciclib treatment, which generally resolved with appropriate dose adjustments; and *venous thromboembolism* (VTE) occurring in ~5–6% of patients treated with abemaciclib. Most patients with AEs were treated with standard anticoagulants and obviously continued treatment with abemaciclib [32,33,34] [Table 1].

It should be noted that patients who develop new (or often worsening) symptoms need to be evaluated for interstitial lung disease/pneumonitis utilizing high-resolution CT, bronchoalveolar lavage, or biopsy, as indicated clinically. Per the label recommendations, complete blood counts and complete metabolic profile should be evaluated at baseline, every 2 weeks (for the first 2 months), monthly (for the subsequent 2 months), and then as necessary clinically [40].

## 10. Conclusions and Future Directions

The efficacy of abemaciclib, a CDK4/6 inhibitor, has been proven, and abemaciclib was well tolerated in breast cancer and endometroid endometrial cancer when combined with aromatase inhibitors in a phase II trial. These results are hypothesis-generating and should be further studied in a phase III trial. Abemaciclib is already FDA-approved for HR+/HER2− breast cancer patients; hence, the future direction should involve testing the combination of abemaciclib with ICIs in patients with endometrial cancer as it has been proven that CDK4/6 inhibitors may promote antitumor immunity and may enhance the action of immune checkpoint inhibitors.

## Figures and Tables

**Table 1:** A summary of reported key adverse events treated with abemaciclib for patients with breast cancer [32,33,34].

Adverse Event	Grades 1–2 (%)	Grade 3 (%)	Grade 4 (%)
Fatigue	20 (67)	1 (3)	0 (0)
Diarrhea	19 (63)	2 (7)	0 (0)
Nausea	11 (37)	1 (3)	0 (0)
Vomiting	6 (20)	0 (0)	0 (0)
Abdominal pain	8 (27)	0 (0)	0 (0)
Neutrophil count ↓	8 (26)	4 (13)	2 (7)
Anemia	10 (34)	4 (13)	1 (3)
Platelet count ↓	8 (27)	2 (7)	0 (0)
WBC ↓	2 (7)	1 (3)	0 (0)
ALT increased	1 (3)	2 (7)	0 (0)
AST increased	3 (10)	1 (3)	0 (0)
Creatinine increased	10 (33)	0 (0)	0 (0)
Urinary tract infection	0 (0)	1 (3)	0 (0)

↓: decrease.
